# Supplementary figures and images for: Transient Global Ischemia-Induced Brain Inflammatory Cascades Attenuated by Targeted Temperature Management
Source: Int J Mol Sci. 2021 May 12;22(10):5114. doi: 10.3390/ijms22105114 (PMC8151768; doi:10.3390/ijms22105114)

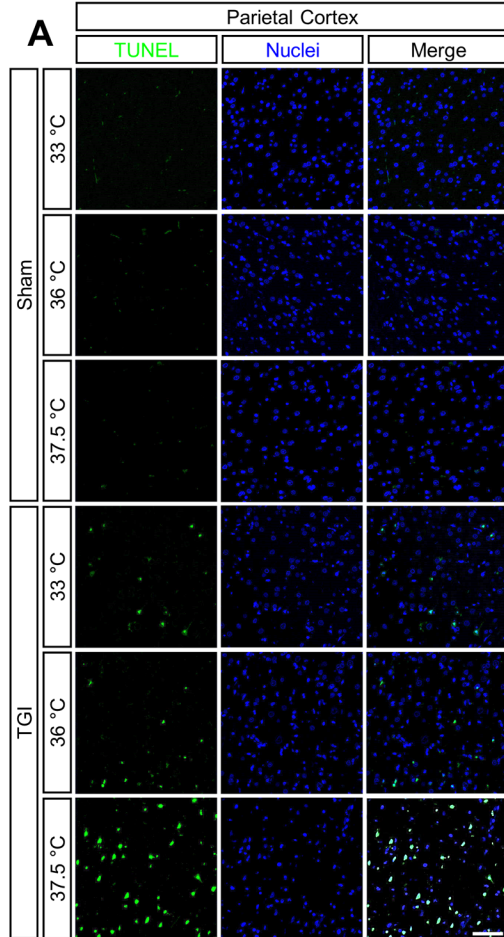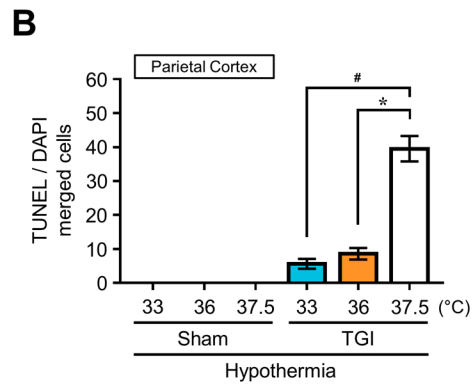

Supplement: Supplementary file 1 [file ijms-22-05114-s001.zip › ijms-1192726-supplementary.pdf]
